# Medical Temperance Association

**Published:** 1893-06

**Authors:** 


### Medical Temperance Association.

This society, organized at the Washington meeting of the American Medical Association, has manifested a vigor and strength that promises to have a very large influence in the future. Founded on the same essential platform as the English Society which has been in existence seventeen years, it aims to rouse up and foster scientific study of the alcoholic question.

The English society numbers several hundred members, who yearly contribute many papers and studies of statistics concerning alcohol and its value as a medicine, and other branches of the same topic. Dr. B. W. Richardson is president, and a journal is issued as an organ of this society, the members of which now include some of the most distinguished medical men of Great Britian. Several branches are in active operation, holding meetings monthly and quarterly, and sending delegates to the annual meeting, which convenes at the same time and place as the British Medical Association. The American society is presided over by Dr. N. S. Davis, and has already over a hundred members. The annual meeting will be at Milwaukee, June 8th, (as the members all belong to the American Medical Association) and will no doubt, attract much attention. In this country, where temperance and the alcoholic question occupy so large a part of the sociological and political topics of the hour, it seems eminently proper for physicians to study and teach the public on this matter. Of all others, medical men are the most competent and have the best facilities for sound counsel in this field. Yet, to-day, almost the entire literature, together with the leaders and teachers of the uses and abuses from alcohol, are a confused medley of statements by the most incompetent persons. It seems startling that the alcoholic question should receive so little attention from physicians, when it is literally a scientific subject which can only be known and understood by the methods of science. This society has begun on this basis. Two prizes of a hundred dollars each are to be awarded to the best essays, "On Alcohol as a Medicine," also on "The Physiological Action of Alcohol on the Body," at the coming meeting. A committee on statistics of the mortality from alcohol, will report on the year's work. Several papers will be read on medical phases of this subject at the coming meeting. This society ignores all political or moral discussions of temperance, and seeks to study the subject from the scientific side. Every medical man in the country should be interested in this work, for notwithstanding all that has been written, the subject is practically unknown; and will be until it is studied generally by physicians.

We take pleasure in calling attention to this society and its work, and believe that as long as they confine their studies exclusively to the scientific side of the alcoholic question, they will fill a very important place in the progress of medicine. The English Society on the same basis, has been very influential in pointing out facts and conclusions concerning alcohol and its influence not known before; and we have no doubt that this society will be equally influential and attain high rank in the years to come. All persons who are interested should write the secretary, Dr. Crothers, of Hartford, Conn., for transactions of last meeting and programs of the coming session.—*Am. Med. Ass'n Journal.*

